# Effect of Zinc Supplementation on Maintenance Hemodialysis Patients: A Systematic Review and Meta-Analysis of 15 Randomized Controlled Trials

**DOI:** 10.1155/2017/1024769

**Published:** 2017-12-31

**Authors:** Ling-Jun Wang, Ming-Qing Wang, Rong Hu, Yi Yang, Yu-Sheng Huang, Shao-Xiang Xian, Lu Lu

**Affiliations:** ^1^The First Affiliated Hospital, Guangzhou University of Chinese Medicine, Guangzhou, Guangdong 510407, China; ^2^Guangzhou Key Laboratory of Chinese Medicine Prevention and Treatment of Chronic Heart Failure, School of Traditional Chinese Medicine, Southern Medical University, Guangzhou 510515, China; ^3^School of Traditional Chinese Medicine, Southern Medical University, Guangzhou 510515, China

## Abstract

We aimed to examine the effects of zinc supplementation on nutritional status, lipid profile, and antioxidant and anti-inflammatory therapies in maintenance hemodialysis (MHD) patients. We performed a systematic review and meta-analysis of randomized, controlled clinical trials of zinc supplementation. Metaregression analyses were utilized to determine the cause of discrepancy. Begg and Egger tests were performed to assess publication bias. Subgroup analysis was utilized to investigate the effects of zinc supplementation in certain conditions. In the crude pooled results, we found that zinc supplementation resulted in higher serum zinc levels (weighted mean difference [WMD] = 28.489; *P* < 0.001), higher dietary protein intake (WMD = 8.012; *P* < 0.001), higher superoxide dismutase levels (WMD = 357.568; *P* = 0.001), and lower levels of C-reactive protein (WMD = −8.618; *P* = 0.015) and malondialdehyde (WMD = −1.275; *P* < 0.001). The results showed no differences in lipid profile. In the metaregression analysis, we found that serum zinc levels correlated positively with intervention time (*β* = 0.272; *P* = 0.042) and varied greatly by ethnicity (*P* = 0.023). Results from Begg and Egger tests showed that there was no significant bias in our meta-analysis (*P* > 0.1). Results of subgroup analysis supported the above results. Our analysis shows that zinc supplementation may benefit the nutritional status of MHD patients and show a time-effect relationship.

## 1. Introduction

Zinc is an essential trace element for humans which is found in nearly 100 specific enzymes. Zinc plays “ubiquitous biological roles” in physiological function, including gene expression, protein synthesis, immune function, and behavioral responses [1–3]. Although the prevalence of zinc deficiency is still unclear in patients with maintenance hemodialysis (MHD), some data show adverse outcomes that may attribute to zinc deficiency [4, 5]. Malnutrition, as an independent risk factor of cardiovascular events, and death are the most common complications observed in MHD patients [6, 7]. Some studies have reported the potential relationship between zinc deficiency and other imbalances, such as oxidative stress, inflammation, or immunosuppression [8, 9], and these disorders may contribute to poor prognosis of disease.

Many previous studies have investigated the effects of zinc supplementation in MHD patients. The relevant results show that zinc supplementation can improve a number of disorders including low-grade inflammatory process, protein-energy wasting, and impaired immune response [10, 11]. However, to our knowledge, there may be some inadequacies in separate clinic trials. For example, in a separate randomized controlled trial (RCT), it is hard to identify the effects of zinc supplementation in MHD patients with different races and varying intervention dosages. The existing data showed different and even contradictory results simultaneously within different studies [12, 13]. Yet, no studies have focused on the causes of heterogeneity. The incomparable evidences make it hard to assess the actual effects of zinc supplementation in MHD patients. These controversies may be ascribed to inadequate data and differences in experimental designs between the published investigations. A meta-analysis may help to find the sources of heterogeneity and clarify the effects of zinc supplementation on nutritional status, oxidative stress, and inflammation.

In this study, we collected relevant RCTs for systematic review and performed meta-analyses to comprehensively investigate the relationships between nutritional status, oxidative stress, and inflammation and zinc supplementation in MHD patients. Subsequently, we aimed to find conflicting results and analyze their causes. Our study may add to the existing literature.

## 2. Methods

### 2.1. Literature Search Strategy

Systematic literature searches were conducted in the following electronic databases: PubMed, Embase, Web of Science, Chinese Biomedical Literature, and the Cochrane Library. The relevant articles were published before January 15, 2016. Only publications with sufficient data were included for assessment. The following search terms were used: [Hemodialysis OR Dialysis OR Renal Replacement Therapy OR End Stage Renal Disease] AND [Zinc OR Zn] AND [Random *∗* OR Randomized Controlled Trial OR Randomized Controlled Trial as Topic] AND [Nutrition OR Nutritional Status OR Reactive Oxygen OR Oxidative Stress OR Inflammation].

To identify additional potentially relevant publications, the related references from all retrieved articles and reviews were manually searched. Only published studies with full-text articles were included in the meta-analysis. All data were entered into the Review Manager 5.0 software (Biostat, NJ, USA) by one author and checked by another author. Any disagreements were resolved by discussion between the two authors and by seeking the opinion of a third party when necessary.

### 2.2. Inclusion and Exclusion Criteria

Types of studies included published reports of RCTs comparing zinc supplementation with controls (placebo or blank control) with available data for outcomes. Type of participants included studies that were restricted to any patients with MHD therapy stability. Records of the basic characteristics of participants (age, sex ratio, and dialysis duration) were required. Type of intervention included studies comparing zinc supplementation with a control for MHD patients. The dose of zinc compounds (zinc sulfate, zinc gluconate, or zinc aspartate) was converted to elemental zinc dose. The control intervention included placebo and blank control.

All outcomes were extracted for type of outcome measures. Only outcomes measured in at least 2 studies were included for the meta-analysis and were as follows: nutritional status: serum zinc levels, body mass index (BMI, kg/m^2^), normalized protein equivalent of nitrogen appearance rate (nPNA, g/kg/d), dietary protein intake (g/kg/d), albumin (g/dL), hemoglobin, triglycerides (g/dL), total cholesterol (mg/dL), low density lipoprotein (mg/dL), and high density lipoprotein (mg/dL) levels; inflammation: C-reactive protein (CRP, ng/mL); and oxidative stress: malondialdehyde (MDA, nmol/mL) and superoxide dismutase (SOD, U/g Hb).

Studies were excluded if there is lack of data regarding oral dosage of zinc or intervention time. Trials of sexual dysfunction were also excluded due to prior analysis. If any studies included multiple publications on the same RCT, we chose the one with the highest quality according to the study quality assessment.

### 2.3. Study Quality Assessment

The method quality of each study was evaluated by 2 authors independently using the Cochrane Risk of Bias Tool that included 6 evaluation criteria [14]: random sequence generation (selection bias), allocation sequence concealment (selection bias), blinding (performance and detection bias), selective outcome reporting (reporting bias), incomplete outcome data (attrition bias), and other potential sources of bias. The judgment for each criterion was indicated as “low risk of bias,” “high risk of bias,” or “unclear risk of bias.”

### 2.4. Data Extraction and Synthesis

Two independent investigators performed data extraction using the inclusion criteria as described above. All discrepancies were resolved by discussion and, if required, participation by a third author. The following information was extracted from each study: first author's surname, year of publication, race and geographical location of the study, sample size, age, base disease, dialysis duration, oral zinc dose, the control intervention (placebo or blank control), and outcomes. The 2 investigators' results were compared, and disagreements were resolved by discussion.

### 2.5. Statistical Analysis

Statistical analyses were performed using Stata version 10.0 software (StataCorp LP, College Station, TX, USA). The effect of each outcome was determined by calculating the respective weighted mean difference (WMD) with a 95% confidence interval (CI). Heterogeneity of the effect size was evaluated using the *Q* and *I*-squared statistics. A fixed effects model was used when the *P* value was >0.05 and *I*-squared was <50%; otherwise, a random effects model was used. The significance of the pooled WMD was determined using a *Z* test. We used Begg and Egger tests to investigate the publication bias of our meta-analysis. To explore the sources of heterogeneity, we performed a meta-regression analysis. Subgroup analyses were also used to evaluate the effect in various conditions. *P* values < 0.05 were considered significant. The above work was completed by 2 authors and checked by a third author.

## 3. Results

### 3.1. Characteristics of Studies

**Figure 1:**
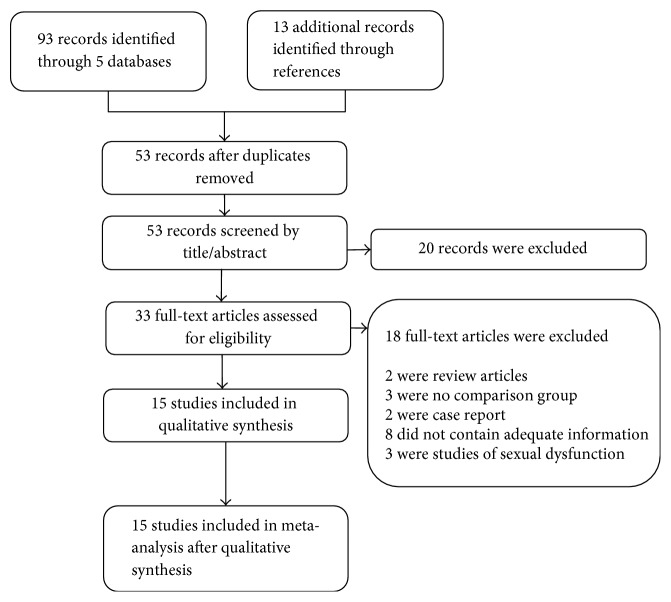
Flow chart of included studies.

A total of 106 relevant published articles were identified following the aforementioned retrieval strategy. After strict review, 91 of these publications were excluded (73 records were screened by title/abstract and 18 records were screened by full-text) and 15 relevant published articles were identified and selected for our meta-analysis (Figure 1). The included studies enrolled a total of 645 MHD patients, among which 345 were treated with zinc supplementation and 300 were treated with placebo or as blank control. Of these included studies, 8 were West Asian (Iran, Turkey, and Egypt), 5 were European or American (United Kingdom, United States, and Mexico), and 2 were East Asian trials (Taiwan and Japan). All studies included patients with chronic kidney disease (CKD) and one complicated low protein catabolic rate. Mean age of participants ranged from 13 to 80 years with dialysis for at least 3 months. The elemental zinc doses ranged from 11 to 100 mg and follow-up ranged from 40 to 360 days. The main characteristics of included studies are summarized in Table 1.

### 3.2. Quality Assessment of Included Studies

**Figure 2:**
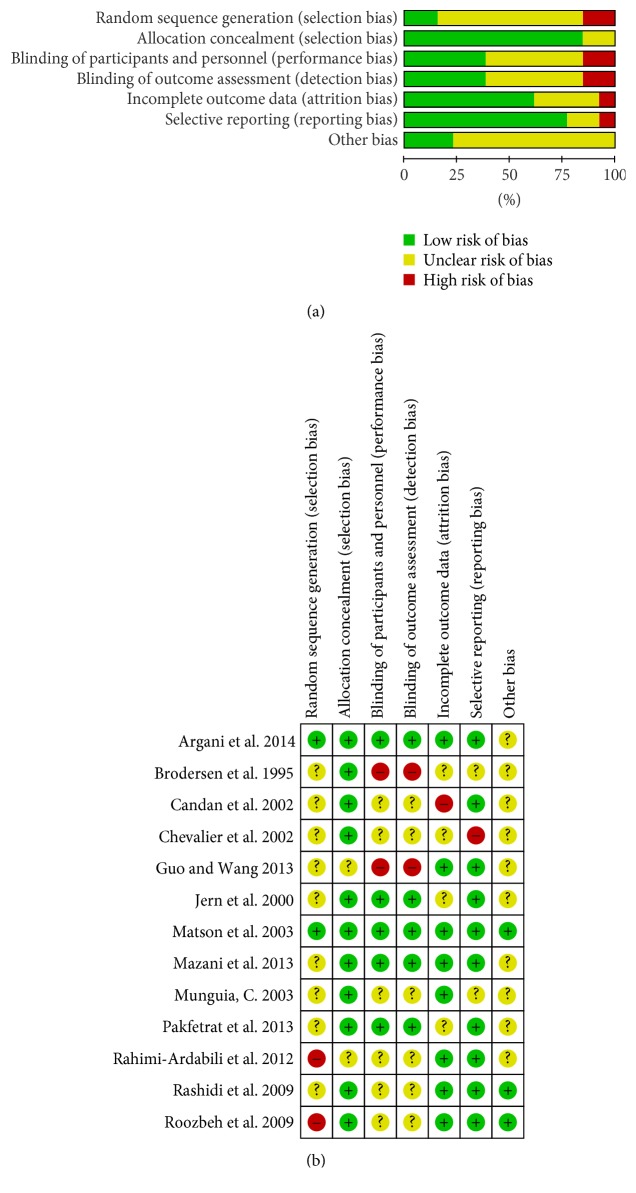
Risk of bias graph and bias summary: (a) review of authors' judgments regarding each risk of bias item for each included study and (b) review of authors' judgments regarding each risk of bias item presented as percentages across all included studies.

Of the 15 included studies, all claimed to apply randomized methods; however, only 2 used such methods (drawing random numbers). One clearly described allocation concealment (third party ensuring). Ten studies had a double-blinded design, but the details were unclear. Only one study described the pharmacy clinical trials unit as a third party, ensuring a double-blinded design. Eight studies reported withdrawals, but the results were not analyzed on an intention-to-treat basis. Of the 15 included studies, 8 reported all expected outcomes. Only 4 studies reported dietary restrictions. This may have caused potential bias due to insufficient information in the included trials. A summary of findings is shown in Figure 2.

### 3.3. Crude Pooled Results of Each Outcome

In the crude analysis, we found that levels of serum zinc, dietary protein intake, and SOD in the zinc supplementation group were higher than levels in control group after treatment. The pooled WMDs were statistically significant (serum zinc: WMD = 28.489, 95% CI = 26.264 to 30.713, *P* < 0.001; dietary protein intake: WMD = 8.012, 95% CI = 1.592 to 14.408, *P* < 0.001; SOD: WMD = 357.568, 95% CI = 152.158 to 562.978, *P* = 0.001). CRP and MDA levels were lower after zinc supplementation (CRP: WMD = −8.618, 95% CI = −15.579 to −1.656, *P* = 0.015; MDA: WMD = −1.275, 95% CI = −1.945 to −0.605, *P* < 0.001). The results showed no differences in BMI, nPNA, hemoglobin, or lipid profile (*P* > 0.05). Heterogeneity was significant in the results. Of the 15 pooled outcomes, 10 showed obvious heterogeneity (*I*-squared > 50%, *P* < 0.1). Results from Begg and Egger tests showed that there was no significant bias in our meta-analysis (*P* > 0.1). All data are presented in Table 2. Relative bioavailability of zinc sulfate, gluconate, and aspartate may be different and worth further analysis. For inadequate reports, meta-analysis for effect of different zinc compounds was not performed.

### 3.4. Results of Metaregression and Region-Subgroup Analysis

**Figure 3:**
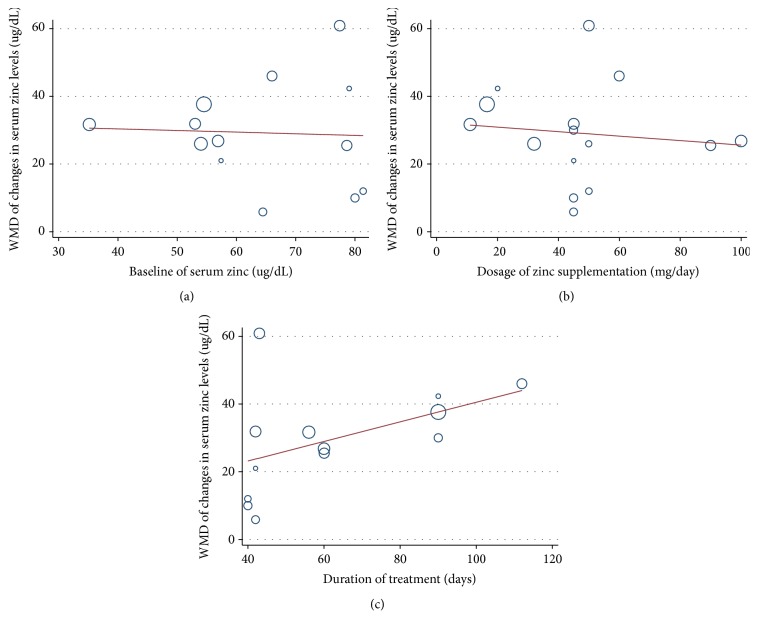
Metaregression data of serum zinc levels based on (a) serum zinc, (b) oral zinc dose, and (c) intervening time at baseline.

**Figure 4:**
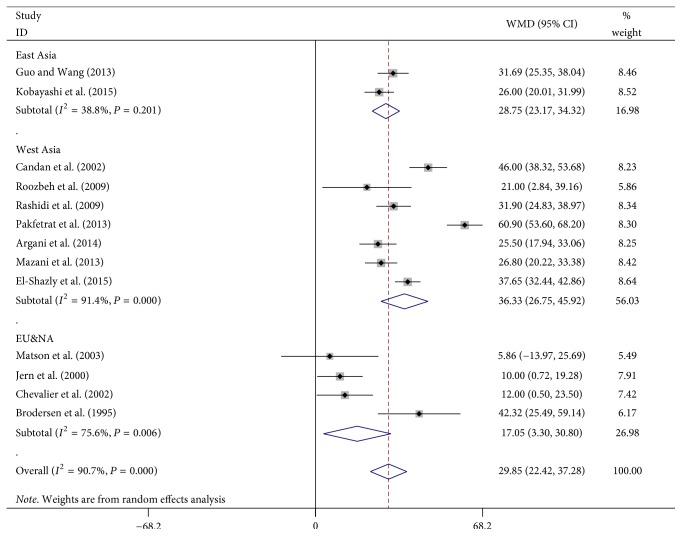
Forest plot: the effect of zinc supplementation on serum zinc levels in different regions (East Asia, West Asia, and Europe/North America).

To explore the sources of heterogeneity, we performed a metaregression analysis on serum zinc levels. Oral zinc dose, intervention time, baseline of serum zinc, and region of study were selected as dependent variables. As shown in Figures 3 and 4, we found that serum zinc levels correlated positively with intervention time (*β* = 0.272, *P* = 0.042). Subgroup data suggested a significant difference among race (*P* = 0.023), and serum zinc levels of patients in Europe and America showed the lowest effect. These 2 factors explained 43.83% of the bias. No correlations were identified between serum zinc levels and oral zinc dose (*β* = −0.066, *P* = 0.691) or baseline zinc levels (*β* = −0.048, *P* = 0.885).

### 3.5. Results of Dose Subgroup

To explore the effects of zinc dose on various outcomes, we performed a dose-special subgroup analysis. The results showed zinc supplement results in higher serum zinc levels and lower CRP and MDA levels, which was consistent with the crude results. However, no dose-effect trend was found when zinc dose changed. In the heterogeneity test, 8 results showed obvious heterogeneity out of a total of 23 (*I*-squared > 50%, *P* < 0.1). All data are presented in Table 3.

### 3.6. Results of Intervening Time Subgroup

To investigate the effects of intervention time on a series of outcomes, we performed a time-special subgroup analysis. In the results, we found that zinc supplementation induced a time effect in serum zinc levels and dietary protein intake. Although our results showed zinc supplementation results in higher SOD levels and lower CRP and MDA levels in all subanalyses, no time effect was identified. Heterogeneity was lower in the time subgroup compared with results in the crude analysis. Of the 25 results analyzed, 4 showed obvious heterogeneity (*I*-squared > 50%, *P* < 0.1). All data are presented in Table 4.

## 4. Discussion

Some dialysis complications, such as malnutrition and inflammation, were partly attributed to zinc deficiency. From previous studies, the conclusions regarding zinc supplementation for MHD patients were not consensus. The effect on nutritional status was one of the most controversial results. Contrary to the majority of opinions and our results, Matson et al. reported that evidence is limited for proving the effects of zinc supplementation on anorexia and nutritional status in MHD patients [12]. An earlier article attributed this discrepancy to age of patients and zinc absorption [28]. Notably, in the study of Matson et al., differences in serum zinc levels were not statistically significant between the treatment and placebo groups. However, in our analysis, we found that zinc supplementation increased serum zinc levels for MHD patients. Our results showed that intervention time and race are two factors that correlated significantly with serum zinc levels, and there is a time-effect but not a dose-effect relationship in zinc supplementation. In the included studies, we found that the median intervention time was 60 days and the median zinc dose was 45 mg/day. In the study of Matson et al., patients received 220 mg zinc sulfate (45 mg elemental zinc) per day for 6 weeks; thus, the limited intervention time may be a possible explanation. It is also plausible that zinc supplementation showed different effects from various racial groups. The mean change of serum zinc levels in the Taiwanese population was several times higher than in the Western regions [8]. European studies showed the lowest effect compared with data from Asia, most likely due to differences in epidemiology and diet. Further studies are warranted to comprehensively investigate the precise effect on different racial groups. Another notable finding was that, although no statistical significance was found in data on albumin and hemoglobin, the *P* values were close to the significance threshold (albumin: *P* = 0.061; hemoglobin: *P* = 0.069). Subgroup analyses also showed a time-effect relationship in the two factors. We assume the long-term effect of zinc supplementation may improve these nutritional indices, but this interpretation also requires further testing. Nonetheless, our results suggest intervention time of zinc supplementation should be adequate when aiming to improve nutritional indices and appetite.

Hypercholesterolemia and hypertriglyceridemia have been reported in previous studies on zinc-deficient diets, which could induce cardiovascular events and insulin resistance in CKD patients [29, 30]. Although a series of previous studies suggest that zinc supplementation improves blood lipid metabolism, this finding was not drawn from our pooled data. The effects and trends of zinc supplementation on lipid profile were inconsistent and even contradictory in the results [17, 20, 21]. There are several possible explanations for this discrepancy. First, it is possible that the characteristics of included patients were divided. For example, zinc supplementation could increase blood lipids by improving energy intake in patients with anorexia [31] and show an opposite effect when including patients with hyperlipemia or insulin resistance [32–34]. Second, little is known about lipid intake from the included studies; diverse dietary lipid intake may have led to information bias. Therefore, more evidence is needed to determine the effects of zinc supplementation on lipid profile in MHD patients.

Inflammation and oxidative stress are common complications in MHD patients. Several previous studies have found that zinc deficiency in MHD patients may result in increased oxidative stress and CRP concentrations [22, 35]. The antioxidative action of zinc involves 2 mechanisms: (1) directly protecting easily oxidized groups such as sulfhydryl and (2) producing some other ultimate long-term antioxidant like metallothionein [36, 37]. Zinc could also decrease CRP and other inflammatory cytokines through increased antioxidant power; the major target is most likely the NF-kappa b pathway [38, 39]. In our analysis, all the included studies showed that zinc supplementation was positive for anti-inflammatory and antioxidant activity. The pooled results showed statistical significance in all subgroups, and no significance for time or dose effect was observed. This may suggest that zinc supplementation may lead to an anti-inflammatory and antioxidative effect in MHD patients.

It should be noted that there might be some possible limitations in our meta-analysis. First, adverse outcomes of zinc supplementation were not analyzed. As is known, although zinc is an essential requirement for good health, an excess in zinc supplementation can be harmful. Excessive absorption of zinc can suppress copper and iron absorption and may cause nerve damage [40, 41]. In the included studies, only one paper mentioned asking patients whether they had experienced any adverse effects. No side effects could be analyzed in the pooled data; incomplete data may have deterred this evaluation. Second, due to insufficient data, the effects of zinc supplementation on clinical endpoint events, such as cardiovascular events or death, remain unclear. Such discrepancies make it difficult to attain strong evidence for MHD patients. Finally, the epidemiology varies significantly across the different regions. For example, zinc deficiency is widespread in East Asia (nearly 40% to 60% of the population have mild or moderate zinc deficiency); the proportion is much lower in Europe and America [42, 43]. However, meta-analyses of the racial subgroups could not be performed due to lack of data in most outcomes. This may have generated a selection bias in our results.

## 5. Conclusion

Our meta-analysis suggests that zinc supplementation benefits the nutritional status of MHD patients and shows a time-effect relationship. It also leads to an anti-inflammatory and antioxidative effect in MHD patients. Still, there is a need for more evidence regarding the effects on lipid profile. Given the presence of data deficiency in this study, further studies are warranted to comprehensively investigate the effects of zinc supplementation on clinical endpoint events and on race.

## Figures and Tables

**Table 1:** Characteristics of included studies.

Study	Reference	Year	Region	Number of patients (M/F)^a^	Age (Year)^b^	Primary disease	Dialysis duration	Elemental zinc dose	Intervening time	Comparative approach	Outcomes^c^
Kobayashi et al.	[15]	2015	Japan	70 (43/27)	69 ± 10	CKD	-	34 mg/day	90/180/270/ 360 days	Blank	Serum zinc, hemoglobin, RBC, ESA, ERI
El-Shazly et al.	[16]	2015	Egypt	30 (29/31)	13.2 ± 2.1	CKD	≥6 months	16.5 mg/day	90 days	Palcebo	Serum zinc, leptin, body weight, BMI
Argani et al.	[17]	2014	Iran	60 (36/24)	(50, 60)	CKD	-^c^	90 mg/day	60 days	Palcebo	Serum zinc, albumin, BMI, body fat, body water, Ccr, hematocrit, hemoglobin, leptin, TC, TG
Pakfetrat et al.	[18]	2013	Iran	97 (55/42)	51.6 ± 16.8	CKD	>3 months	50 mg/day	43 days	Placebo	Serum zinc, HCys, homocysteine
Mazani et al.	[19]	2013	Iran	65 (41/24)	52.7 ± 12.6	CKD	>6 months	100 mg/day	60 days	Placebo	Serum zinc, BMI, GSH, MDA, SOD, TAC
Guo and Wang	[8]	2013	Taiwan	65	59.7 ± 9.2	CKD	>3 months	11 mg/day	56 days	Blank	Serum zinc, hematocrit, albumin, CD4/CD8, CRP, GFR, IL-6, MDA, nPNA, Cu, SOD, TNF-*α*, Vit C/E
Rahimi-Ardabili et al.	[20]	2012	Iran	60 (38/22)	52.7 ± 12.7	CKD	≥6 months	100 mg/day	60 days	Placebo	TC, Apo-AI, Apo-B, HDL, LDL, PON, TG
Roozbeh et al.	[21]	2009	Iran	53 (28/25)	55.7	CKD	≥6 months	45 mg/day	42 days	Placebo	Serum zinc, HDL, LDL, TC, TG
Rashidi et al.	[22]	2009	Iran	55 (32/23)	57.6	CKD	≥6 months	45 mg/day	42 days	Placebo	Serum zinc, CRP, hemoglobin
Nava-Hernandez and Amato	[23]	2005	Mexico	25	16.6	CKD	-	100 mg/day	90 days	Placebo	Albumin, pre-albumin, transferrin
Matson et al.	[12]	2003	UK	15 (11/4)	63.73	CKD	≥3 months	45 mg/day	42 days	Placebo	Serum zinc, albumin, Kt/V, calcium, CRP, nPNA, phosphate
Chevalier et al.	[24]	2002	USA	27 (22/6)	51.9	CKD	≥6 months	50 mg/day	40/90 days	Placebo	Serum zinc, dietary intake, HDL, LDL, TC
Candan et al.	[25]	2002	Turkey	34 (18/16)	45.6 (28, 64)	CKD	-	20 mg/day	90 days	Placebo	Serum zinc, MDA, OSMO fragility
Jern et al.	[26]	2000	USA	14	56.5 (23, 80)	CKD with low PCR	≥6 months	45 mg/day	40/90 days	Placebo	Serum zinc, dietary intake, nPNA
Brodersen et al.	[27]	1995	German	40 (22/18)	60	CKD	-	60 mg/day	112 days	Blank	Serum zinc

*Note*. ^a^Sex ratio: M = male, F = female; ^b^age appears as mean, mean ± standard deviation or mean (lower limit, upper limit); ^c^-: no information was recorded in included study. *Abbreivations*. RBC, red blood cell; ESA, erythropoiesis-stimulating agent; ERI, ESA resistance index; BMI, body mass index; Ccr, creatinine clearance rate; TC, total cholesterol; TG, triglyceride; HDL, high-density lipoprotein; LDL, low-density lipoprotein; CRP, C-reactive protein; GFR, glomerular filtration rate; MDA, malondialdehyde; nPNA, normalized protein equivalent of nitrogen appearance; SOD, superoxide dismutase.

**Table 2:** Summary of the effects of zinc supplementation in MHD patients.

Factor	Number of studies	Heterogeneity test			Weighted mean difference			Publication bias	
		*Q*	*I* ^2^	*P* value	WMD	[95% CI]	*P* value	Begg test	Egger test
Serum zinc (ug/dL)	13	81.98	90.7%	<0.001	28.489	[26.264, 30.713]	<0.001^*∗*^	0.502	0.355
BMI (kg/m2)	3	1.34	0%	0.511	0.149	[−0.762, 1.059]	0.794	1.000	0.263
nPNA (g/kg/d)	4	11.31	91.20%	0.001	0.135	[−0.161, 0.431]	0.371	1.000	0.657
Dietary protein intake (g/kg/d)	2	0.27	0%	0.604	8.012	[1.592, 14.408]	<0.001^*∗*^	-	-
Albumin (g/dL)	4	9.74	69.20%	0.021	0.358	[−0.016, 0.732]	0.061	0.734	0.276
Hemoglobin (g/dL)	4	16.27	81.6%	0.013	0.756	[−0.011, 1.522]	0.053	1.000	0.654
HDL (mg/dL)	4	24.6	91.90%	<0.001	4.048	[−3.142, 11.238]	0.27	1.000	0.847
LDL (mg/dL)	4	24.46	91.80%	<0.001	21.028	[−15.478, 57.534]	0.259	1.000	0.749
TC (mg/dL)	5	22.97	86.90%	<0.001	16.198	[−9.975, 42.371]	0.225	0.734	0.624
TG (mg/dL)	3	8.2	75.60%	0.017	0.207	[−34.711, 35.125]	0.991	1.000	0.327
CRP (ng/mL)	3	13.85	85.60%	0.001	−8.618	[−15.579, −1.656]	0.015^*∗*^	1.000	0.783
MDA (nmol/mL)	3	16.32	87.70%	<0.001	−1.275	[−1.945, −0.605]	<0.001^*∗*^	0.296	0.287
SOD (U/g Hb)	2	3.42	70.80%	0.064	357.568	[152.158, 562.978]	0.001^*∗*^	-	-

*Note*. -: values could not be calculated due to an insufficient number of studies; ^*∗*^*P* < 0.05, and WMD was considered statistically significant.

**Table 3:** Results of dose subgroup.

Factor	Zinc dose	Number of studies	Heterogeneity test			Weighted mean difference		
			*Q*	*I* ^2^	*P* value	WMD	[95% CI]	*P* value
Serum zinc (ug/dL)	<45 mg	4	2.27	27.52%	0.211	30.792	[23.781, 44.201]	<0.001^*∗*^
	45–50 mg	6	121.15	95.90%	<0.001	23.831	[4.824, 42.837]	0.014^*∗*^
	>50 mg	3	18.01	88.90%	<0.001	32.692	[20.111, 45.273]	<0.001^*∗*^
BMI (kg/m2)	16.5 mg	1	-	-	-	0.530	[−3.769, 2.709]	0.621
	≥90 mg	2	1.16	13.70%	0.282	0.124	[−1.062, 1.309]	0.838
nPNA (g/kg/d)	11 mg	1	-	-	-	0.41	[0.292, 0.528]	<0.001^*∗*^
	45 mg	2	1.3	23.00%	0.250	0.019	[−0.130, 0.167]	0.805
Dietary protein intake (g/kg/d)	45 mg	2	0.64	0.00%	0.425	5.605	[−0.527, 11.736]	0.073
	50 mg	2	0.53	0.00%	0.467	5.373	[−1.351, 12.097]	0.117
Albumin (g/dL)	<50 mg	2	6.96	85.60%	0.008	0.37	[−0.225, 0.966]	0.223
	≥50 mg	2	2.35	57.40%	0.125	0.309	[−0.343, 0.962]	0.353
Hemoglobin (g/dL)	<45 mg	2	3.62	71.87%	0.054	1.018	[−0.188, 2.223]	0.098
	≥45 mg	2	0.08	0.00%	0.780	0.385	[−0.307, 1.078]	0.275
HDL (mg/dL)	<50 mg	2	31.35	96.80%	<0.001	4.083	[−14.455, 22.621]	0.666
	≥50 mg	2	10.09	96.80%	<0.001	0.031	[−6.494, 6.556]	0.993
LDL (mg/dL)	<50 mg	2	4.84	79.40%	0.028	6.088	[−25.371, 37.548]	0.704
	≥50 mg	2	0.46	0.00%	0.496	44.792	[34.951, 54.632]	<0.001^*∗*^
TC (mg/dL)	≤50 mg	2	0.03	0.00%	0.857	37.045	[26.472, 47.617]	<0.001^*∗*^
	>50 mg	2	1.81	44.70%	0.179	−2.381	[−19.731, 14.968]	0.788
TG (mg/dL)	<50 mg	2	7.67	87.00%	0.006	0.337	[−53.525, 54.200]	0.99
	≥50 mg	1	-	-	-	−3.112	[−40.718, 34.718]	0.876
CRP (ng/mL)	11 mg	1	-	-	-	−5.799	[−8.925, −2.673]	<0.001^*∗*^
	45 mg	2	2.11	52.60%	0.146	−10.234	[−20.861, −0.392]	0.039^*∗*^
MDA (nmol/mL)	<50 mg	2	10.34	90.30%	0.001	−1.617	[−2.948, −0.286]	0.017^*∗*^
	≥50 mg	1	-	-	-	−0.8	[−0.995, −0.605]	<0.001^*∗*^

*Note*. -: values could not be calculated due to an insufficient number of studies; ^*∗*^*P* < 0.05, and WMD was considered statistically significant.

**Table 4:** Results of intervention time subgroup.

Factor	Intervention time	Number of studies	Heterogeneity test			Weighted mean difference		
			*Q*	*I* ^2^	*P* value	WMD	[95% CI]	*P* value
Serum zinc (ug/dL)	<50 days	6	21.15	85.90%	<0.001	23.831	[4.824, 42.837]	0.014^*∗*^
	50–60 days	4	1.82	0.00%	0.402	28.310	[24.399, 32.220]	<0.001^*∗*^
	>60 days	5	1.07	12.90%	0.388	36.065	[25.694, 46.437]	<0.001^*∗*^
BMI (kg/m2)	60 days	2	1.16	13.70%	0.282	0.124	[−1.062, 1.309]	0.838
	90 days	1	-	-	-	0.530	[−3.769, 2.709]	0.621
nPNA (g/kg/d)	<50 days	2	0.58	0.00%	0.447	−0.018	[−0.128, 0.092]	0.751
	≥50 days	2	1.3	31.10%	0.243	0.235	[−0.108, 0.578]	0.179
Dietary protein intake (g/kg/d)	<50 days	2	0.18	0.00%	0.776	3.322	[−3.407, 9.407]	0.359
	≥50 days	2	0.12	0.00%	0.812	8.109	[1.592, 14.408]	<0.001^*∗*^
Albumin (g/dL)	<60 days	2	1.96	45.60%	0.134	0.301	[−0.225, 0.966]	0.223
	≥60 days	2	2.17	47.40%	0.125	0.409	[−0.243, 1.062]	0.353
Hemoglobin (g/dL)	<60 days	2	0.04	0.00%	0.850	0.378	[−0.048, 0.804]	0.082
	≥60 days	2	3.69	72.90%	0.055	1.171	[0.083, 2.259]	0.035^*∗*^
HDL (mg/dL)	<60 days	2	31.35	96.80%	<0.001	4.083	[−14.455, 22.621]	0.666
	≥60 days	2	10.09	90.10%	0.001	0.031	[−6.494, 6.556]	0.993
LDL (mg/dL)	<60 days	2	1.96	49.00%	0.161	34.829	[17.061, 52.597]	<0.001^*∗*^
	≥60 days	2	24.46	95.90%	<0.001	19.971	[−36.680, 76.623]	0.490
TC (mg/dL)	<60 days	2	1.5	33.20%	0.221	18.673	[−2.741, 40.088]	0.087
	≥60 days	3	22.15	91.00%	<0.001	11.147	[−18.788, 41.082]	0.465
TG (mg/dL)	<60 days	1	-	-	-	26.080	[6.602, 45.558]	0.009
	≥60 days	2	1.01	8.60%	0.317	−17.415	[−42.743, 7.913]	0.178
CRP (ng/mL)	<60 days	2	2.11	14.60%	0.146	−10.234	[−20.861, 0.392]	0.059
	≥60 days	1	-	-	-	−5.799	[−8.925, −2.673]	<0.001^*∗*^
MDA (nmol/mL)	<60 days	1	-	-	-	−2.330	[−3.048, −1.612]	<0.001^*∗*^
	≥60 days	2	0.53	0.00%	0.467	−0.831	[−1.007, −0.654]	<0.001^*∗*^
SOD (U/g Hb)	<60 days	1	-	-	-	446.600	[340.276, 552.924]	<0.001^*∗*^
	≥60 days	1	-	-	-	234.200	[35.906, 432.494]	0.021^*∗*^

*Note*. -: values could not be calculated due to an insufficient number of studies; ^*∗*^*P* < 0.05, and WMD was considered statistically significant.
